# Does carbon dioxide gas-induced pneumothorax and one-lung ventilation really have similar oncological outcomes in minimally invasive esophagectomy?

**DOI:** 10.1097/JS9.0000000000001036

**Published:** 2024-01-08

**Authors:** Kexun Li, Jie Mao, Yunchao Huang

**Affiliations:** Department of Thoracic Surgery I, Key Laboratory of Lung Cancer of Yunnan Province, Yunnan Cancer Hospital, The Third Affiliated Hospital of Kunming Medical University, Cancer Center of Yunnan Province, Kunming, People’s Republic of China

*Dear Editor,*

We have read with great interest a paper titled ‘Carbon dioxide gas-induced pneumothorax versus one-lung ventilation in minimally invasive esophagectomy: A multicenter propensity score matching cohort study’ published in the *International Journal of Surgery* by Chen *et al*.^1^. In this study, the authors conducted a large, multicenter cohort study and utilized univariate analysis along with propensity score matching (PSM) and inverse probability of treatment weight (IPTW) to adjust for confounding factors. While Chen and colleagues have conducted a well-matched and meaningful study, there are certain points that require further discussion.

Firstly, according to current guidelines and expert consensus, endoscopic submucosal dissection (ESD) is considered an optimal treatment strategy for AJCC staging stages Tis and T1a^2,3^. However, the study included a total of 152 patients with stage Tis (15.2%) and 151 patients with T1 (15.1%) who underwent minimally invasive esophagectomy (MIE). This limitation in samples may impact the study’s findings and conclusions.

Secondly, the significance of preoperative therapy in patients with T2/T3N0-1 esophageal squamous cell carcinoma has been recognized in the CROSS study. This finding was further emphasized in the NEOCRTEC5010 study and is also mentioned in current guidelines^3,4^. However, the study included 648 patients with T2 or T3 stages (68.5%) and 204 patients with N1 stages (20.4%) who did not receive preoperative therapy. The study did not specify the TNM stage of the patients; it is unclear whether some stage II–III patients are without neoadjuvant therapy.

Lastly, the present study is an interesting research in which more detailed baseline information and clinicopathological characteristics should be available to readers so as to be more helpful in guiding clinical practice. For example, the ‘Methods’ section mentioned cancer history was not put in the relevant tables. The patient’s physical condition also has a certain impact on the survival outcomes, and relevant scores such as the KPS score or EOCG score should be supplemented. Moreover, some studies have shown that lymphadenectomy can also affect survival outcomes^5^. Only minimally invasive McKeown esophagectomy with extended 2-field lymph node dissection was mentioned in this article without the number of lymph node dissection. These indicators affecting patient survival should be mentioned in the tables to improve the credibility of the study’s conclusion.

## Ethics approval

None.

## Sources of funding

This work was supported by the National Natural Science Foundation of China (81960335), the National Clinical Key Specialty Construction Project (Thoracic surgery construction project), and the Science and Technology Department of Yunnan Province (Yunnan Province of Li Yin specialist workstation).

## Author contribution

K.L., J.M., and Y.H.: study concept and design, acquisition, analysis, or interpretation of data; K.L.: drafting of the article and statistical analysis; Y.H. and J.M.: administrative, technical, or material support and obtained funding; K.L., J.M., and Y.H.: had full access to all the data in the study and take responsibility for the integrity of the data and the accuracy of the data analysis.

## Conflicts of interest disclosure

The authors have no conflicts of interest to declare.

## Research registration unique identifying number (UIN)

None.

## Guarantor

Kexun Li and Yunchao Huang are guarantors.

## References

[1] Chen J Heng J Zheng B . Carbon dioxide gas-induced pneumothorax versus one-lung ventilation in minimally invasive esophagectomy: a multicenter propensity score matching cohort study. Int J Surg 2023. doi:10.1097/JS9.0000000000000968. [Epub ahead of print]. PMC10942216 38051934

[2] Libanio D Pimentel-Nunes P Bastiaansen B . Endoscopic submucosal dissection techniques and technology: European Society of Gastrointestinal Endoscopy (ESGE) Technical Review. Endoscopy 2023;4:361–389. 10.1055/a-2031-0874 36882090

[3] Ajani JA D’Amico TA Bentrem DJ . Esophageal and esophagogastric Junction Cancers, Version 2.2023, nccn clinical Practice guidelines in Oncology J Natl Compr Canc Netw 2023;4:393–422. 10.6004/jnccn.2023.0019 37015332

[4] Yang H Liu H Chen Y . Neoadjuvant chemoradiotherapy followed by surgery versus surgery alone for locally advanced squamous cell carcinoma of the esophagus (NEOCRTEC5010): a phase III multicenter, randomized, open-label clinical trial. J Clin Oncol 2018;27:2796–2803. 10.1200/JCO.2018.79.1483 PMC6145832 30089078

[5] Li K Leng X He W . Resected lymph nodes and survival of patients with esophageal squamous cell carcinoma: an observational study. Int J Surg 2023;7:2001–2009. 10.1097/JS9.0000000000000436 PMC10389544 37222685

